# Mixed effects of complement in a chronic murine model of inflammatory erosive arthritis and pulmonary vascular disease

**DOI:** 10.1371/journal.pone.0340677

**Published:** 2026-02-05

**Authors:** Kiana L. Chen, Stacey Duemmel, Michael Christof, Gaochan Wang, H. Mark Kenney, Marc Nuzzo, Qingfu Xu, Benjamin Korman, Homaira Rahimi

**Affiliations:** 1 Department of Pathology and Laboratory Medicine, University of Rochester Medical Center, Rochester, New York, United States of America; 2 Center for Musculoskeletal Research, University of Rochester Medical Center, Rochester, New York, United States of America; 3 Department of Medicine, Division of Allergy, Immunology, and Rheumatology, University of Rochester Medical Center, Rochester, New York, United States of America; 4 Department of Pediatrics, University of Rochester Medical Center, Rochester, New York, United States of America; Kansai Medical University: Kansai Ika Daigaku, Institute of Biomedical Science, JAPAN

## Abstract

Complement’s role in the pathology of rheumatoid arthritis and pulmonary hypertension (PH) is not fully understood. We aimed to determine whether complement deficiency is protective against joint and lung disease using the tumor necrosis factor-transgenic (TNF-Tg) mouse model characterized by chronic inflammatory-erosive arthritis and PH. TNF-Tg synovium and lungs were analyzed with bulk and single-cell RNA-sequencing. TNF-Tg mice were crossed with complement component 3 knockout (C3KO) and factor B knockout (fBKO) mice to quantify disease. Knee histology was scored, CD45 + Ly6C-C5aR1 + cells were quantified by flow cytometry, mid-hindpaw bone volumes were determined with micro-computed tomography, and lung disease was quantified with histology and right heart catheterization. TNF-Tg mice have upregulated complement-related genes, however, C3 and fB knockout did not cause significant changes in pathology. Synovium from C3KO TNF+ mice had a greater C5aR1 + cell population than TNF-Tg mice (*p < 0.0001*). In early disease, C3KO TNF+ mice exhibited greater bone volumes (*p < 0.05, p < 0.01)* than other TNF+ mice. Complement deficiency in a chronic inflammatory model does not ameliorate joint or pulmonary disease. However, C3 deficiency may delay complement-dependent bone erosions. The influence of complement on disease may change with persistent inflammation. Chronic TNF-mediated disease may represent a pathologic endotype that is differentially mediated by complement.

## Introduction

The complement system is a vital component of innate immunity, with roles that include targeting pathogens to fight against infection, recruiting and regulating immune cells, and clearing immune complexes and apoptotic cells in tissue [1–3]. Protein components of the complement system circulate throughout the body and can activate immune processes during infection and autoimmunity. Component 3 (C3) is a major protein of the complement system and is responsible for the early activation and persistence of the complement cascade by aiding in the cleavage and recruitment of other complement proteins. C3 hydrolysis produces C3(H_2_O), a component of the C3 convertase complex that cleaves C3 into C3a and C3b which serve important effector functions in both the classical and alternative complement pathways [1–4]. Factor B (fB) is a complement protein specific to the function of the alternative pathway, as it is a component of C3 and C5 convertase in this pathway and necessary for their production [2,3]. The production and function of components in the differing pathways dictates the activity of the complement cascade and influence on immune processes. In autoimmune diseases, such as systemic lupus erythematosus (SLE), rheumatoid arthritis (RA) and systemic sclerosis (SSc), there is dysregulation and inadequate control of the complement system, with evidence of complement component consumption or increased split products which are thought to contribute to pathogenesis [1]. It is also known that genetically regulated lack of certain complement proteins is associated with development of autoimmune disease [5].

In RA, disease progression is characterized by complex mechanisms that lead to the dysregulation of the immune system. Genetic and environmental factors stimulate autoreactivity that recruits immune cells to infiltrate the joint, upregulate inflammatory-related gene expression, and increase immune cell activation [6–8]. These pathways lead to chronic inflammatory signaling in the joint which progresses to the generation of a hyperplastic synovium, pannus formation, and the destruction of cartilage and bone [9,10]. In inflammatory arthritis, the complement cascade has significantly increased activity in response to immune complexes that form in the synovial tissue, recruiting immune cells to the joint and contributing to the inflammatory milieu [1,11–13]. More recently, it has been recognized that intracellular complement, particularly in synovial fibroblasts, plays an important role in tissue priming [14]. Complement system proteins have also been found to contribute to bone erosion by positively regulating osteoclast formation, with evidence suggesting that complement specifically contributes to increased osteoclastogenesis in inflammatory disease [15,16]. Similarly, studies of acute inflammatory arthritis with complement deficient mice (i.e., C3, fB, and C5), have shown associated protection from collagen-induced arthritis (CIA), collagen antibody-induced arthritis (CAIA), and serum transfer arthritis [17–19]. Complement proteins have been found to have increased expression and activity in the cartilage, synovial fluid, and synovial tissue of RA patients where these proteins can act as inflammatory mediators alongside cytokines to recruit and stimulate inflammatory immune cells that cause joint disease [1,11–13]. Additionally, recent clinical studies have also examined a decrease in complement activity and expression with RA treatment [20,21]. This suggests that complement could serve as a potential therapeutic target in RA.

SSc is an autoimmune disorder characterized by autoimmunity, vasculopathy, and fibrosis. Pulmonary hypertension (PH) is characterized by pulmonary vascular remodeling and perivascular inflammation and is a leading cause of SSc morbidity and mortality [22]. SSc patients with prominent vascular disease have evidence of microvascular complement deposition, and we have previously shown that patients with SSc associated PH have elevated levels of serum adipsin (complement factor D) [23]. In an experimental system of hypoxia induced PH, C5 and the alternative complement cascade were critical for PH induction, and immunoglobulins mediated activation of the complement cascade [24]. Whether complement plays a pathogenic role in SSc associated PH is unknown.

Tumor necrosis factor-transgenic (TNF-Tg; line 3647) mice represent a model of both inflammatory-erosive arthritis and PH. Specifically, these mice demonstrate synovitis and bone erosion as well as perivascular lung inflammation, occlusive pulmonary vasculopathy, and elevated right ventricular pressures [25–27]. Given that TNF is critical to pathogenesis, the joint and lung disease is caused initially by innate immune dysregulation that then leads to a chronic and progressive disease phenotype, which is in contrast to acute and inducible models of arthritis and PH. Therefore, we aim to determine if the complement cascade may play a distinct role in disease in the TNF-Tg model, as it models diseases that do have changes in complement signaling to increase inflammation and disease pathology. This investigation has important implications since this model recapitulates important aspects of human disease, including remarkable sexual-dimorphism with early onset and severe disease in females compared to males [26]. Given that we previously showed that these animals have a significant increase in C5aR1^+^ (C5a receptor 1^+^) lung macrophages [28], we hypothesized that alterations in complement may be important in driving TNF-induced pathology in both arthritis and pulmonary hypertension. To investigate the role of complement in the pathogenesis of these disorders, we crossed TNF-Tg mice with mice lacking C3 (all complement pathways) and fB (lacking alternative pathway activation) to determine whether complement deficiency would be protective of joint or lung pathology.

## Materials and methods

### Ethics statement

This study does not use human participants or human participants data.

All animal research and procedures were previously approved by the University of Rochester Medical Center University Committee for Animal Resources (UCAR). Cohorts of mice that underwent live micro-computed tomography imaging were scanned under anesthesia using a 1–3% isoflurane concentration with oxygen. Mice were determined to be properly induced using the toe pinch method and were monitored for complete recovery after imaging. Cohorts of mice that underwent right heart catheterization were also induced with isoflurane anesthesia, placed on a warmed platform for the duration of the procedure, and were euthanized after surgery with an anesthetic overdose using injectable ketamine. Other euthanasia methods throughout the study included carbon dioxide euthanasia and was followed by cervical dislocation as a secondary method.

### Animals

The 3647 line of TNF-Tg mice overexpress one copy of the human TNF-ɑ gene [25], and were originally obtained from Dr. George Kollias (Institute of Immunology, Alexander Fleming Biomedical Sciences Research Center, Vari, Greece) [29,30]. Wildtype (WT, TNF-) littermates were used as controls.

C3-deficient mice are homozygous for the deletion of the complement component 3 gene. The C3 knockout (C3KO) mice were originally obtained by Jackson Laboratory (JAX stock #003641) [31]. TNF-Tg mice were bred with C3KO mice to generate C3KO and C3KO TNF-Tg mice used in this study. Similarly, fB knockout (fBKO) mice are homozygous for the deletion of the complement factor B gene have been previously described [32]. TNF-Tg mice were bred with fbKO mice to generate fbKO and fbKO TNF-Tg mice used for this study. In the remainder of this manuscript, cohorts with the TNF gene will be labeled TNF+ and without will be labeled TNF-. Littermate controls were used in all experiments. The cohorts compared in this study were bred at the same time and had their data collected and analyzed simultaneously.

All mice were bred on a C57BL/6 genetic background. As we have previously shown that female TNF-Tg mice demonstrate an earlier and more severe joint and lung pathology with mortality by 5–6-months of age [26], female mice were used for these experiments. Genotypes were verified by standard PCR in which DNA is extracted from tissue, added to a PCR master mix containing buffers, salts, dNTPs, DNA polymerase, and primers, amplified in a PCR heat cycle, and run through a 2% agarose gel with SYBR safe DNA gel stain (ThermoFisher, S33102) (TNF and C3). fBKO genotypes were also verified with a TaqMan based automatic system from Transnetyx (Transnetyx, Cordova, Tennessee) in which DNA is extracted and purified from tissue then used in a real-time PCR reaction with a labeled probe specific for the gene of interest. The following primers were used for TNF-Tg genotyping: Forward primer: 5’ TACCCCCTCCTTCAGACACC 3’ and reverse primer: 5’ GCCCTTCATAATATCCCCCA 3’. The following primers were used for C3KO genotyping: Common: 5’ ATCTTGAGTGCACCAAGCC 3’; Wildtype: 5’ GGTTGCAGCAGTCTATGAAGG 3’; and Mutant: 5’ GCCAGAGGCCACTTGTGTAG 3’. The following primers were used for fBKO genotyping: Cfb KO forward primer: 5’ GACCGCAGGACTTTGAAAATGG 3’; Cfb KO reverse primer: 5’ CAGACTGCCTTGGGAAAAGC 3’; Cfb WT forward primer: 5’ GACCGCAGGACTTTGAAAATGG 3’; Cfb WT reverse primer: 5’ AAATCTGGTCACTCAGGTTGTAGAAG 3’. The reporter sequencing for Cfb KO is 5’ ACCCGGTAGAATTC 3’ and the reporter sequence for Cfb WT is 5’ ATTCTGGCCCCGGTCCC 3’.

### Bulk RNA-sequencing

Lungs were isolated from 4-month-old TNF- (n = 3) and TNF+ (n = 3) female mice. Total RNA was extracted from lungs using a RNeasy Plus Micro Kit (Qiagen, Valencia, CA). and subjected to RNA-sequencing using an Illumina HiSeq2500v4 high-throughput DNA sequencer at the University of Rochester Genomics Research Center. The TruSeq Stranded mRNA Sample Preparation Kit (Illumina, San Diego, CA) was used for next generation sequencing library construction. Briefly, mRNA was purified from 200ng total RNA with oligo-dT magnetic beads and fragmented. First-strand cDNA synthesis was performed with random hexamer priming followed by second-strand cDNA synthesis using dUTP incorporation for strand marking. End repair and 3` adenylation was then performed on the double stranded cDNA. Illumina adaptors were ligated to both ends of the cDNA and amplified with PCR primers specific to the adaptor sequences to generate cDNA amplicons of approximately 200–500 bp in size. The sequencing depth was 50-60M reads per sample. Sequenced reads were cleaned according to a pre-processing workﬂow (Trimmomatic-0.32) before mapping them to the Mus musculus genome (mm10) with STAR version 2.5.2b. Subsequently, deSeq2 was used to perform differential gene expression analysis of uniquely mapped transcripts with an FDR cutoff of 0.05 (95% conﬁdence interval) and enrichR was used for pathway analysis [33–35].

### Single-cell RNA-sequencing

Single-cell suspensions were obtained from the lungs and synovium of TNF+ and TNF- mice (n = 3–5 per genotype in lung, 5–10 per genotype in synovium) by a combination of mechanical dissociation and enzymatic digestion. Tissue was pooled by group and dissociated with GentleMACS Octo Dissociator (Miltenyi Biotec) followed by enzymatic digestion with Collagenase A (COLLA-RO, Roche), Elastase (Worthington, ESL LS002292) and Dispase II (Corning, 354235) for lung and type II collagenase (Worthington, CLS-2 LS004174) for synovium. Enzymatic digestion of tissue was performed in a 37°C incubator with rotation (16x-21x) and vortex (every 15 minutes) functions for 45–60 minutes. Single cells were washed using ice-cold PBS and collected at 300xg for 5 minutes. Red blood cells were removed using ACK lysis buffer (Invitrogen, A1049201. Subsequently, viable cells were sorted using LIVE/DEAD Fixable Aqua Dead Cell Stain Kit (Invitrogen, L34965). 100,000–300,000 cells were collected and submitted for single cell capture. Cellular suspensions were loaded on a Chromium Single-Cell Instrument (10x Genomics, Pleasanton, CA, USA) to generate single-cell Gel Bead–in–Emulsions. Single-cell RNA-seq libraries were prepared using Chromium Single-Cell 3′ Library & Gel Bead Kit (version 1.1; 10x Genomics). The beads were dissolved, and cells were lysed per the manufacturer’s recommendations. Samples were loaded on a Chromium Single-Cell Instrument (10x Genomics, Pleasanton, CA, USA) to generate single-cell GEMs (Gel Bead-in-Emulsions). GEM reverse transcription (GEM-RT) was performed to produce a barcoded, full-length cDNA from poly-adenylated mRNA. After incubation, GEMs were broken, the pooled GEM-RT reaction mixtures were recovered, and cDNA was purified with silane magnetic beads (DynaBeads MyOne Silane Beads, ThermoFisher Scientific). The purified cDNA was further amplified by PCR to generate sufficient material for library construction. Enzymatic fragmentation and size selection was used to optimize the cDNA amplicon size and indexed sequencing libraries were constructed by end repair, A-tailing, adaptor ligation, and PCR. Final libraries contain the P5 and P7 priming sites used in Illumina bridge amplification. Libraries were sequenced using Illumina’s NovaSeq6000 instrument. 5000–8000 cells were captured per condition at a read depth of 50,000 reads per cell. The sequencing reads were examined by quality metrics, the raw FASTQ reads were aligned to the 10X genomic reference mouse genome (refdata-gex-mm10–2020-A) using Cell Ranger (version 7.0.1) (10 × Genomics) count function with default parameters. Data analysis was performed using R (version 4.2.3). Seurat 5.0.1 was used for initial data analysis, normalization of gene expression, integration of samples, and identification and visualization of cell populations using UMAP projections. Dimension reduction, clustering, and analysis of data were performed in Seurat using the default settings. Cells with expression of less than 200 and greater than 5000 genes, and cells with greater than 20% expression of mitochondrial genes were filtered out of analysis. The integration of the sequencing datasets was performed using the R package Harmony. Cell populations were identified by using the FindConservedMarkers function and cell annotation was performed using the Tabula Muris database and the cell markers at LungMAP for the lung data [36,37].

### Histology

Mice were euthanized at 2.5-, 3.5- and 4.5-months of age and the knee joints collected and fixed in 10% neutral buffered formalin for 24–48 hours (n = 3–8 mice/cohort). Knee samples subsequently underwent decalcification for two weeks using a 14% EDTA standard decalcification solution. Tissue was then rinsed in distilled water twice, then transferred to 50% ethanol for 15 minutes before undergoing paraffin processing with the Sakura Tissue Tek VIP6 automated tissue processor. Paraffin processing included tissue being cycled through a series of increasing ethanol concentrations for dehydration, three cycles of xylene at ambient temperature, and four cycles of paraffin at 58°C for one hour in each cycle. The pressure/vacuum function was also on for each cycle to help the reagent infiltrate the tissue. Tissue was then embedded then sectioned (5 µm) onto slides at the University of Rochester Center for Musculoskeletal Research Histology Core Facility. Knee sections were stained with hematoxylin and eosin (H&E) or tartrate-resistant acid phosphatase (TRAP) and counterstained in 0.08% Fast Green. Stained slides were scanned with an Olympus VS120 at 40X magnification and were scored in a blinded manner on a semi-quantitative scale, as previously described [26] in order to grade the severity of synovial inflammatory infiltrate, pannus invasion, and bone erosion. Synovial inflammatory infiltrate and pannus invasion scores were used to determine the severity of joint inflammation. The TRAP score was used to quantify bone erosion as TRAP stains osteoclast cells that can resorb bone [38]. TRAP scoring is determined by the scale of positive TRAP staining on the surface of the bone and was previously used when examining inflammatory erosive disease [26].

Lungs from 3.5-4.5-month-old euthanized female mice were inflated with 10% neutral buffered formalin and harvested. Tissue was fixed in 10% neutral buffered formalin for 2 days, paraffin processed using the same method as the knee samples, embedded in paraffin, and cut into 5 µm sections on microscope slides. Slides were then stained with H&E and scanned at 20X magnification. The adventitia perimeter and lumen/vessel area ratio of pulmonary vessels was determined as a measure of PH, as previously described [27].

### Flow cytometry

Knee joints and lungs of 4-month-old female mice were collected for flow cytometric analysis (n = 7–8 mice/cohort). The knee joints and right lobes of the lung were first manually digested using a scalpel. Knee joints were then incubated with 1U/mL of Liberase (Sigma-Aldritch, 5401020001) in RPMI 1640 buffer with 10% FBS at 37°C for 1.5–2 hours and lung tissue was incubated with 0.2 mg/mL Liberase and 0.1 mg/mL DNase I (Sigma-Aldritch, 10104159001) for 30 minutes. After digestion, homogenized knee joints and lung samples were filtered with 70µm nylon filters to collect single cell suspensions. Lung cells were then suspended in RBC lysis buffer (Thermo Fisher, 00-4333-57) for 5 minutes, then resuspended in PBS. Knee joint cells were also resuspended in PBS. Live cells from all samples were counted on a hemocytometer and 1–8 x 10^6^ cell population was harvested from each sample for staining.

Cells were washed and resuspended in serum-free buffer with 1:1000 LIVE/DEAD Fixable Aqua Stain (Thermo Fisher, L34965) as a viability stain and incubated on ice for 30 minutes. Cells were then washed with staining buffer and blocked with 5 µg/mL Fc Block (clone S17011E; BioLegend, 156604) for 10 minutes before being stained with an antibody cocktail of C5aR1-APC (clone 20/70; BioLegend, 135808), CD45-BV786 (clone 30-F11; BD Biosciences, 564225), and Ly6C-BV421 (clone AL-21; BD Biosciences, 562727) for 30 minutes on ice. Compensation beads (Thermo Fisher, A10628, 01-2222-42) were utilized for single-stained and unstained controls.

Data acquisition was performed on an 18-color BD FACSAria II with BD FACSDiva software in the University of Rochester Medical Center Flow Cytometry Core. Data analysis and evaluation was performed with FCS Express software (V7.18.0021, De Novo Software, USA).

### Micro-CT imaging and analysis

Hindpaws of 2.5- and 4-month-old female mice (n = 4–6 paws/cohort) were collected and imaged with the micro-computed tomography (micro-CT) scanner, VivaCT 40 (Scanco USA, Inc.) at the University of Rochester Medical Center, Center for Musculoskeletal Research’s Biomechanics, Biomaterials, and Multimodal Tissue Imaging Core. Scans were performed for 30–45 minutes with an integration time of 300ms, 2048 x 2048 matrix size, 1000 projections over 180 degrees, and scanned at a 10.5µm resolution. DICOMs were exported to Amira software (V2020.2, Thermo Fisher Scientific, USA) for analysis with a semi-automated segmentation method [39]. The total bone volumes of the cuboid, navicular and lateral intermediate cuneiform, and talus bones were compared between groups.

### Hemodynamic assessment of right ventricular pressure

4.5-month-old female mice underwent right heart catheterization (n = 3–6 mice/cohort). Right ventricle systolic pressure (RVSP), and right ventricle (RV) systolic function (dP/dT) measurements were performed in spontaneously breathing mice under 2.0%–2.5% isoflurane anesthesia delivered in 100% oxygen with a flow of 2.0 L/min by inserting a 1.4F microtip transducing catheter (SPR-1000, Millar Instruments, Houston, TX) via the right jugular vein (closed chest), as previously described [27]. The data was collected with Lab Chart Pro on a PowerLab acquisition device (ADInstruments, Colorado Springs, CO, Cardiovascular Research Institute Surgical Core, University of Rochester Medical Center).

### Statistics

Statistics were performed in GraphPad Prism (V9.5.1, GraphPad Software, USA). Data was analyzed with a one-way ANOVA with Tukey’s post-test for multiple comparisons between groups. Continuous data using parametric analysis were confirmed to have normal distribution with Shapiro-Wilk tests. Outliers, defined as values lesser or greater than 2 standard deviations from the mean, were excluded from analysis. Values reported are mean ± standard deviation.

## Results

### Complement pathway genes are differentially expressed in TNF+ synovial and lung cells

**Fig 1:**
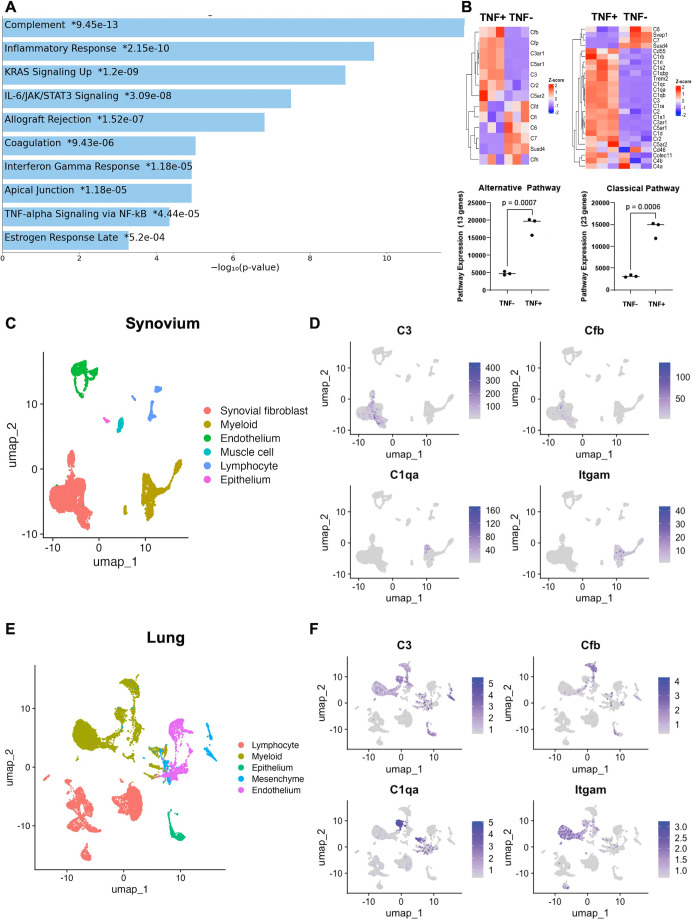
Complement genes are differentially expressed in TNF+ tissue and in specific cell types. Lung tissue of TNF- and TNF+ mice were harvested for bulk RNA-sequencing. **(A)** Pathway expression analysis show complement as the most differentially expressed pathway in cells. **(B)** Heatmaps illustrate the differential expression of specific complement genes between TNF- and TNF+ lung samples collected for bulk-RNA sequencing, with significant expression of alternative and classical complement genes in TNF+ samples. Differential gene expression was performed using the DESeq2 R package, and pathway analysis was performed with enrichR [33–35]. Relative expression is shown in the heatmap and in the pathway expression comparison. The synovium and lung tissue from TNF- and TNF+ mice were harvested for single-cell RNA-sequencing. TNF- and TNF+ datasets were combined to produce the uniform manifold approximation projection (UMAP) visualization for synovium and lung. **(C and E)** UMAPs display different clusters and their cell types. **(D and F)** UMAPs that display the expression patterns of *C3*, *Cfb*, *C1qa*, and *Itgam* were also generated. *Itgam* was used as a pan-marker for myeloid cells. All analysis was done in the Seurat package in R.

Pathway analysis of bulk RNA-sequencing data demonstrated that complement was the most differentially expressed pathway in TNF+ lungs (Fig 1A). Genes associated with the complement pathway (*C3, C3ar1, Cfb, C5ar1, Cr2,* and *C1qa*) were differentially expressed in TNF+ mice in comparison to TNF- mice (Fig 1B). To further characterize the specific expression of complement genes in inflammatory arthritis and PH, and to determine the cells involved in these differences, synovial and lung tissue from TNF+ and TNF- mice were harvested, digested, and processed for single-cell RNA-sequencing (Fig 1C, 1E). While myeloid, lymphocyte, endothelium, and epithelium cells were found in both the synovium and lung, *C3* and *Cfb* were primarily restricted to fibroblasts in the synovium (Fig 1D) and in the lung, these genes were mostly seen in myeloid, endothelium, and epithelial cells (Fig 1F).

### Complement deficiency does not inhibit synovial inflammation in TNF+ mice

**Fig 2:**
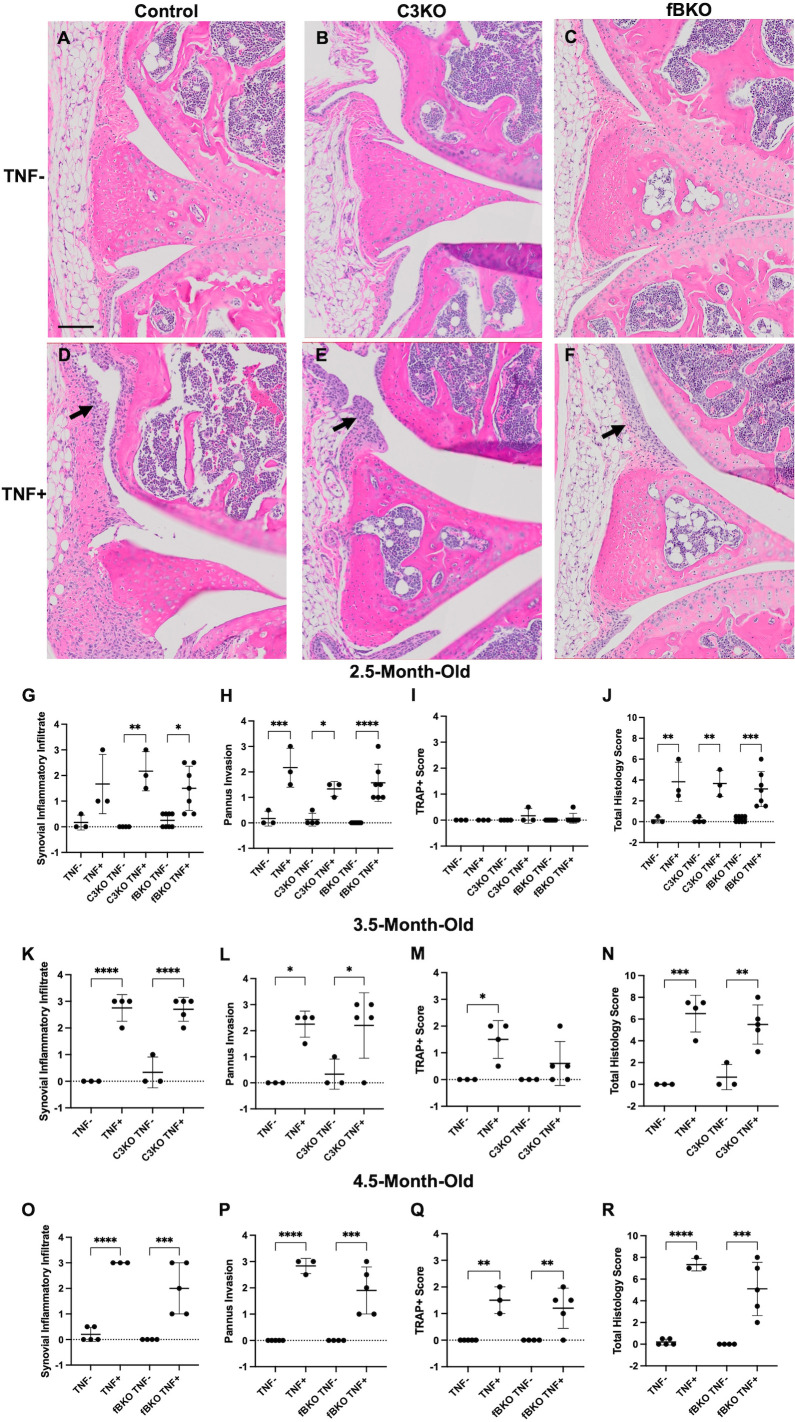
Complement Deficiency Does Not Inhibit Synovial Inflammation in TNF+ Mice. **(A-F)** Representative images of H&E-stained knee joints (40X magnification) from 2.5-month-old TNF-, TNF + , C3KO TNF-, C3KO TNF + , fbKO TNF-, and fbKO TNF+ show synovial inflammation present in TNF+ mice (arrows). Cohorts were scored on synovial inflammatory infiltrate, pannus invasion, and TRAP+ area from TRAP-stained slides at different terminal timepoints. Total histology score is a composite of these scores. **(G-J)** Analysis was completed for all cohorts at 2.5-months-old. **(K-N)** C3KO TNF-, C3KO TNF + , and controls were scored at 3.5-months-old and **(O-R)** fBKO TNF-, fBKO TNF + , and controls were scored at 4.5-months-old. There was no significant difference between TNF+ groups. Analysis was performed with a one-way ANOVA with Tukey’s multiple comparisons. n = 3-8 mice/cohort. * = *p < 0.05*, ** = *p < 0.01*, *** = *p < 0.001*, and **** = *p < 0.0001*. Significant comparisons are not shown between TNF+ and TNF- cohorts that are not their littermate controls. Black scale bar = 100µm.

We crossed specific complement KO mice with the TNF-Tg mice in order to assess the effect of complement on synovial and lung pathology. Representative H&E-stained knee joints of 2.5-month-old females demonstrate synovial inflammation occurring in the TNF+ mice (Fig 2A-2F). As previously described [26], stained slides of knee joints were scored semi-quantitatively to compare the severity of arthritis between control cohorts and the C3KO and fBKO mice at different timepoints. At 2.5 months old, there was a significant difference found between C3KO TNF- and C3KO TNF+ cohorts and fBKO TNF- and fbKO TNF+ cohorts in synovial inflammatory infiltrate. There was also a significant difference between TNF- and TNF+ cohorts in the pannus invasion and total histology score, with increased severity in the TNF+ groups, although not in the TRAP score for each group (Fig 2G-2J). However, there was no difference in the total histology or subsection scores when comparing the TNF+ group with C3KO TNF+ or fbKO TNF+ mice. This was also evident at age 3.5 months (Fig 2K-2N) and at 4.5 months (Fig 2O-2R). Additionally, the TRAP score worsened between control and TNF+ groups as the mice aged, except at 3.5 months C3KO TNF+ (Fig 2M, 2Q).

### C5aR1 + populations in the C3KO TNF+ synovium are significantly greater than populations in the TNF+ mice

**Fig 3:**
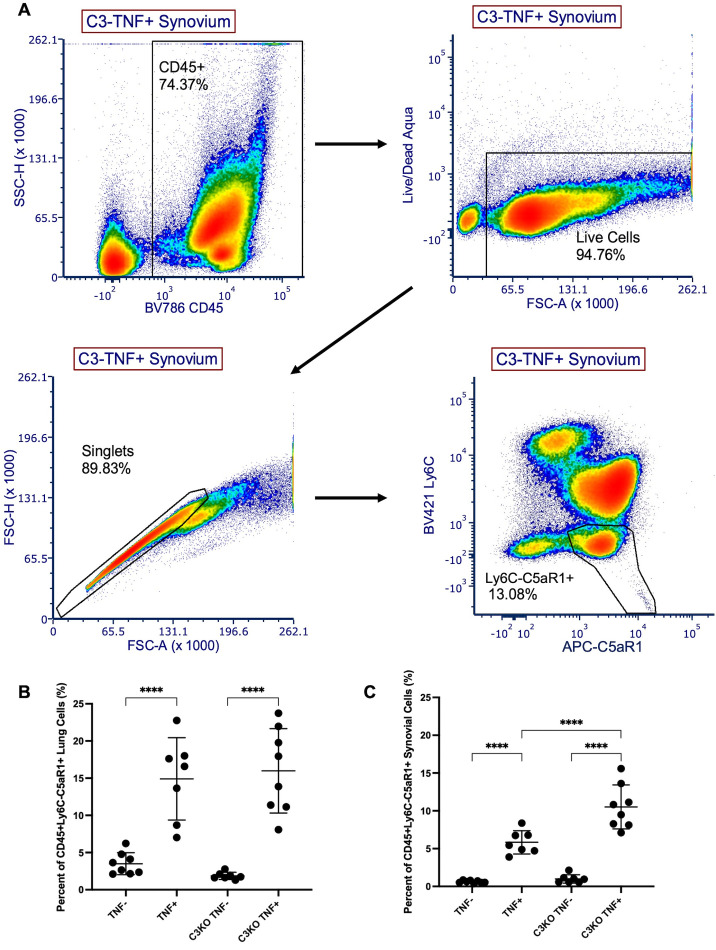
CD45 + Ly6C-C5aR1 + populations in the C3KO TNF+ synovium are significantly greater than populations in TNF+ mice. Synovium and lung tissue were collected from 4-month-old female C3KO TNF+ and C3KO TNF- mice and controls and single cells were stained for CD45, Ly6C, C5aR1, and Live/Dead Aqua. **(A)** The representative gating strategy was used for all samples to quantify live CD45 + Ly6C-C5aR1 + cells. The percentage of CD45 + Ly6C-C5aR1 + cells out of the total count for each sample was compared between groups in **(B)** lung and **(C)** synovium. Analysis was performed with a one-way ANOVA with Tukey’s multiple comparisons. n = 7-8 mice/cohort. **** = *p < 0.0001*. Significant comparisons are not shown between TNF+ and TNF- cohorts that are not their littermate controls.

Single cells harvested from 4-month-old TNF-, TNF + , C3KO TNF-, and C3KO TNF+ synovium and lung were gated for CD45 + Ly6C-C5aR1 + live cells (Fig 3A) and the percentage of these cells out of the total count was compared between cohorts. Increased percentages of C5aR1 + cells were found in the TNF+ groups between TNF- and TNF+ mice and C3KO TNF- and C3KO TNF+ mice in both the lung (Fig 3B) and synovium (Fig 3C). Interestingly, C3KO TNF+ mice had the largest population of CD45 + Ly6C-C5aR1 + cells in the synovium (TNF+ = 5.833 ± 1.544%, C3KO TNF+ = 10.52 ± 2.909%, *p < 0.0001*). Given the association of C5aR1 with bone development and osteoclastogenesis [15,16], we explored the bone phenotype of the cohorts using imaging.

### Bone loss is delayed in C3KO TNF+ mice at early-stage arthritis

**Fig 4:**
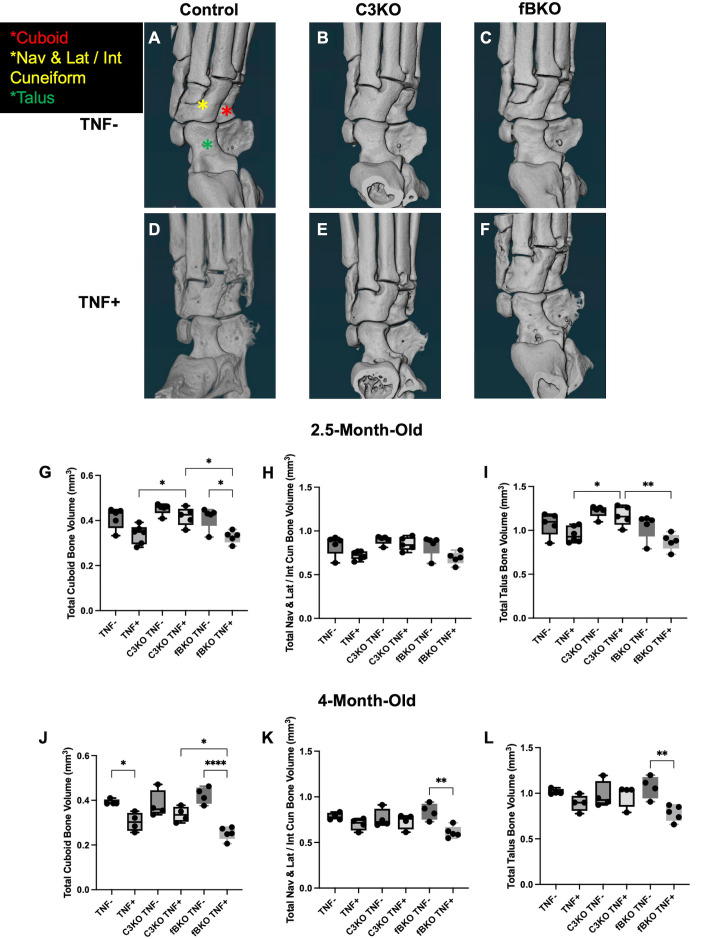
Bone Loss is Delayed in C3KO TNF+ Mice. **(A-F)** Representative images of the 3D rendered micro-CT ankle scans from 2.5-month-old TNF-, TNF + , C3KO TNF-, C3KO TNF + , fBKO TNF-, and fBKO TNF+ mice show the cuboid (red asterisk), navicular and lateral intermediate cuneiform (yellow asterisk), and talus (green asterisk) bones that were used for bone volume comparisons. Cohorts were compared at **(G-I)** 2.5-months-old and **(J-L)** 4-months-old. TNF+ and fBKO TNF+ mice had significantly less bone volume than C3KO TNF+ mice in the cuboid and talus bones at 2.5-months-old, and fBKO TNF+ mice had significant less bone volume than C3KO TNF+ mice in the cuboid bone at 4-months-old. Analysis was performed with a one-way ANOVA with Tukey’s multiple comparisons. n = 4-6 hindpaws/cohort. * = *p < 0.05* and ** = *p < 0.01*, and **** = *p < 0.0001*. Significant comparisons are not shown between TNF+ and TNF- cohorts that are not their littermate controls. There were no significant differences between the TNF- cohorts.

Micro-CT scans of the ankles were performed on all cohorts at early and late stages of disease and analyzed with a previously validated segmentation method [39] to quantify erosive disease seen in TNF+ mice [25,26,40]. Representative images of the 3D-rendered micro-CT scans (Fig 4A-4F) display the cuboid (Fig 4A, red asterisk), navicular and lateral intermediate cuneiform (Fig 4A, yellow asterisk), and talus (Fig 4A, green asterisk) bones that were used to compare bone volume between cohorts. The TNF+ mice scans demonstrate erosive bone loss occurring at the ankle (Fig 4D-4F). All cohorts were analyzed at early-stage disease at 2.5-months-old (Fig 4G-4I) and late-stage disease at 4-months-old (Fig 4J-4L). Notably, C3KO TNF+ mice had significantly greater total bone volume, indicating less bone erosion, than TNF+ and fbKO TNF+ mice in the cuboid (TNF+ = 0.3390 ± 0.04, C3KO TNF+ = 0.4181 ± 0.04, fBKO TNF+ = 0.3279 ± 0.03 mm^3^, *p < 0.05*) and talus (TNF+ = 0.9523 ± 0.09, C3KO TNF+ = 1.1670 ± 0.11, fBKO TNF+ = 0.8728 ± 0.09 mm^3^, *p < 0.05*, *p < 0.01*) bones at 2.5 months (Fig 4G and 4I). At 4 months, the C3KO TNF+ mice had a significantly greater bone volume than the fBKO TNF+ mice (C3KO TNF+ = 0.3366 ± 0.03, fBKO TNF+ = 0.2518 ± 0.03, *p < 0.05*) in the cuboid bone. Additionally, the total bone volume of C3KO TNF+ mice was not significantly different from C3KO TNF- mice, unlike TNF+ and TNF- mice in the cuboid and the fBKO TNF+ mice and fBTNF- mice in all the analyzed bones (Fig 4J-4K). There were no significant differences between TNF+ and fBKO TNF+ mice in any bones at either timepoint.

### C3 and fB deficiency does not protect against pulmonary hypertension in TNF+ mice

**Fig 5:**
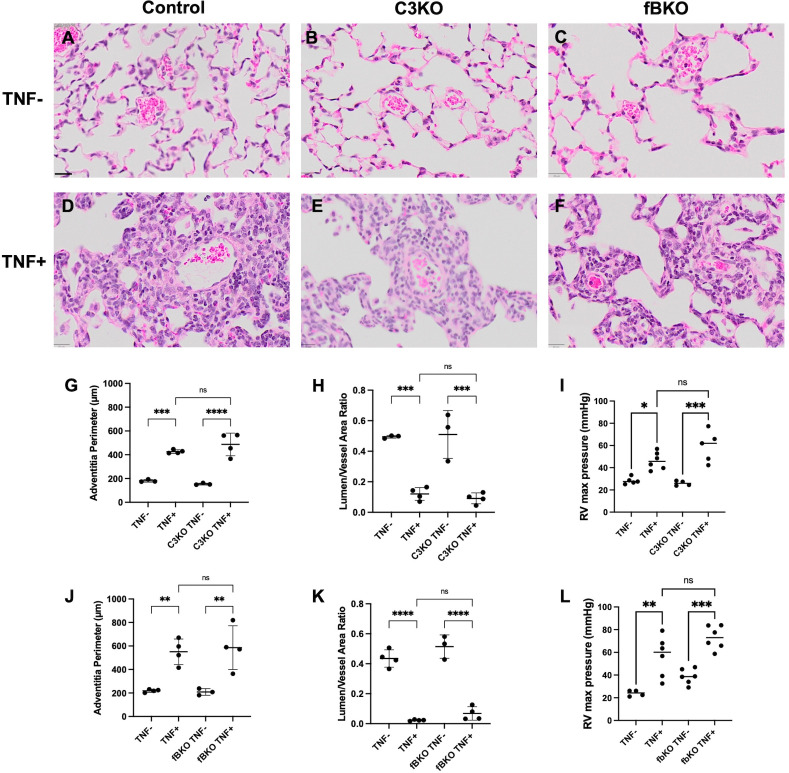
C3 and fB Deficiency Does not Protect Against Pulmonary Disease. **(A-F)** Representative images of H&E-stained lungs (20X magnification) from 3.5-4.5-month-old TNF-, TNF + , C3KO TNF-, C3KO TNF + , fBKO TNF-, and fBKO TNF+ mice are shown. **(G-H and J-K)** The adventitia perimeter and lumen/vessel ratio of the lung was determined by histology and **(I and L)** the right ventricular (RV) pressure was determined by right heart catheterization at 3.5-4.5-months-old. There was no significant difference between TNF+ mice and C3KO TNF+ or fBKO TNF + . Analysis was performed with a one-way ANOVA and Tukey’s multiple comparisons. n = 3-6 mice/cohort. ns = Not significant, * = *p < 0.05*, ** = *p < 0.01*, *** = *p < 0.001*, **** = *p < 0.0001*. Significant comparisons are not shown between TNF+ and TNF- cohorts that are not their littermate controls. Black scale bar = 20µm.

Pulmonary vascular disease was quantified with histopathology analysis of lung tissue and assessment of right ventricular pulmonary pressure [27]. H&E-stained lungs from 4.5-month-old mice revealed vascular occlusion in TNF+ cohorts (Fig 5A-5F). The adventitia perimeter was increased, and the lumen/vessel area ratio was decreased in TNF+ mice compared to the TNF- mice, but there was no significant difference between TNF+ and C3 or fB deficient TNF+ mice (Fig 5G-5H and 5J-5K). Additionally, right heart catheterization measured RV max pressure in all cohorts and showed no significant difference between TNF + , C3KO TNF + , and fbKO TNF+ mice (Fig 5I and 5L). There was also no change in dP/dT measures between cohorts (data available in the public repository).

## Discussion

We investigated the role of classical and lectin (via complement protein C3) and alternative (via fB protein) complement pathways in the progression of both chronic arthritis and connective-tissue disease associated PH using the TNF+ mouse. The complement system and its dysfunction in autoimmunity has been exhibited in the pathology of RA and SSc vascular disease [11–13,24,41]. Genomic studies demonstrated significant differential expression of complement genes in the lung and synovium (Fig 1), leading to the hypothesis that genetic ablation of complement would ameliorate joint and lung pathology in the TNF+ mouse. Our data show that while neither C3 or fB deficiency is protective from inflammatory arthritis or pulmonary hypertension, C3 deficiency may delay bone loss in the TNF+ model.

In acute inducible models of arthritis, the complement pathway, and specifically the alternative pathway, has been shown to be critical to disease induction and severity. Genetic knockout of C3 and fB have significantly ameliorated the severity of synovial inflammation in CIA, CAIA, and K/BxN serum transfer arthritis [17–19]. In CIA mice, histologic analysis revealed there was a protective effect against inflammation and bone erosion with C3 and fB deficiency [17]. Histology of CAIA mice with knockout of C3 also showed significant decrease in arthritis severity and bone erosion [18]. Another group found that when K/BxN serum was injected into C5, C5aR, fB, and C3 knockout mice, ankle thickening from arthritis was significantly reduced [19]. Unlike these models, the TNF-Tg mouse demonstrates chronic inflammation from constitutive human TNF-ɑ production, and the knockout of C3 or fB does not affect the progression or severity of synovial inflammation as measured by histology or imaging parameters. Interestingly, however, there does appear to be an effect on inflammatory-erosive bone disease, though it is not sustained. Examination of the TRAP scores for the knees in each cohort illustrated that bone erosion increased in TNF+ cohorts as mice aged, with significant differences found between TNF- and TNF+ mice at 3.5 months old, and TNF- and TNF+ mice and fBKO TNF- and fBKO TNF+ mice at 4.5 months old (Fig 2). However, there was no notable difference between C3KO TNF- and C3KO TNF+ mice at 3.5 months old, suggesting that bone erosion may not be as severe with C3 deficiency.

It is well established that components of the complement pathway are involved in bone homeostasis and C3 and C5 are produced by osteoblasts and osteoclasts, with their respective receptors found on both types of bone cells [42]. C3 signaling has been found to increase osteoclastogenesis in inflammatory environments by directly stimulating osteoclast formation, increasing the expression of RANKL, and priming synovial fibroblast to be pro-inflammatory and drive osteoclast differentiation [43,44]. Knockout of the complement protein C3a’s receptor, C3aR, has also been described to significantly diminish osteoclastogenesis, suggesting that the loss of C3-related signaling in the C3KO model can also limit osteoclast formation and erosive disease [45]. In the complement system, C3 is also necessary to form the enzyme that cleaves C5 and produces C5a. Notably, C5a has been found to still be generated in the absence of C3 in a model of acute lung injury [46], however C5a expression has not been determined in a chronic inflammatory model and may be significantly decreased with C3 depletion. C5a can recruit inflammatory cells, stimulate pro-inflammatory responses, and aid in the development of immune cells that can contribute to disease, including Th17 cells [47,48]. C5a receptor 1 (C5aR1) signaling with C5a is involved in stimulating inflammation and osteoclast differentiation, as determined by studies in which knockout of C5aR1 leads to decreased osteoclastogenesis [16,43,49,50]. Additionally, recent work using single cell analysis of osteoclasts suggests C5aR1 may be a marker of osteoclast precursor fate [51]. Prior work from our group found an increased C5aR1 + cell population in the lung tissue of TNF+ mice [28]. We validated this finding in our current study by investigating C5aR1 expression in a broad population of immune cells that can respond to C5aR1 signaling and drive inflammatory disease [52,53] (Fig 3) in the lung, and also noted that this C5aR1 + population was present in the synovium of TNF+ mice at age 4 months. Our analysis of single-cell RNA-sequencing in TNF- and TNF+ synovium determined that C5aR1 is primarily expressed in the myeloid population, with expression of the osteoclast marker genes, *Acp5* and *Ctsk* also found in this cluster in TNF+ mice (data available in NIH GEO database). Furthermore, we found an increased population of C5aR1 + cells in the C3KO TNF+ cohort compared to the TNF+ group in our flow cytometry analysis. However, micro-CT imaging revealed that C3KO TNF+ mice have greater bone volume than TNF+ mice in early disease (Fig 4). At 4 months, there was also no significant difference in bone volume between C3KO TNF- and C3KO TNF+ mice in any of the bones. In comparison, fBKO TNF+ mice had significantly lower bone volume than fBKO TNF- mice in each analyzed bone and TNF+ mice had significantly lower bone volume than TNF- mice in the cuboid bone. In prior studies, cohorts of TNF+ mice also had significantly lower bone volume than TNF- mice in multiple other bones [40,54]. This suggests that C3KO TNF+ erosive disease is not as severe as other TNF+ cohorts and that there is mediation of inflammation-induced osteoclastogenesis in the C3KO TNF+ group. Lack of C5aR1 activation can significantly reduce osteoclastogenesis and may possibly affect the expression of C5aR1 + cells. Further studies of C3 and C5 inhibition of TNF-induced erosive disease are necessary to confirm this potential downstream effect and delineate the etiology.

Regarding synovitis, the differential expression of complement which we identified occurs predominantly in the fibroblast and the myeloid cell subsets. Recent work has shown that Thy1 + CD34 + sublining synovial fibroblasts overexpress complement components, and this leads them to undergo metabolic rewiring and subsequent inflammatory tissue priming [14]. It therefore makes sense that models in which arthritis is induced acutely may be more amenable to improvement if complement deficiency can prevent fibroblast priming, but priming may be less relevant in a system of chronic exposure to a pro-inflammatory cytokine like TNF-ɑ.

In the lung, SSc-PAH is associated with elevated levels of complement factor D, a critical regulator of the alternative pathway [23,55], and we have also shown highly differentially expressed complement genes in the lungs of the TNF+ mouse. While we expected that complement would play a more important role, the histology and right heart catheterization data suggest that complement loss-of-function (either the entire cascade or the alterative pathway) does not protect from PH in this context (Fig 5). In a spontaneous altitude induced bovine model of PH, C3 has been shown to play a role in perivascular inflammation by inducing the release of extracellular vesicles which stimulate macrophage activation [56]. Given that our single-cell analysis demonstrates that much of the complement over-expression happens in fibroblasts, this raises the question as to whether there may be a role for intracellular complement (which has also been shown in RA) and whether genetic ablation of the cascade is able to appropriately recapitulate this phenotype.

Disease heterogeneity is an important concept in both RA and SSc with molecular subsets of disease identified at the tissue level (pathotypes [57] or CTAPs in RA [58], intrinsic subsets in SSc [59] frequently corresponding to patients with different pathobiology and likely different treatment responses. In RA, for example, some synovial biopsies demonstrate a marked enrichment of T and B cells while others have a very different cellular makeup with myeloid, fibroblast, and endothelial cells but largely lacking lymphocytes. Given the highly different events inciting arthritis and PH onset in the TNF-Tg model relative to the acute/inducible models where complement has previously shown a role in arthritis and PH, we suggest that TNF+ mice may better represent a fibroblast/myeloid predominant subset of human disease whereas many of the acute models may represent a more lymphocyte predominant inflammation with a prominent role for autoantibodies and that these differences may account for the disparity in the role of the complement cascade. The contrast between the lack of a complement effect in the TNF-Tg model and the protective effect of complement deficiency in acute and inducible RA and PH models gives credence to the importance of using multiple complementary models with different mechanisms that recapitulate molecular subsets to fully appreciate the role of a given pathway in complex diseases like RA and SSc-PAH. In fact, eculizumab and the C5aR inhibitor PMX-53 were both unsuccessful in clinical trials of RA [60], and it would be interesting to examine patient heterogeneity at the molecular level to see whether there may have been a response in a subset of patients. Given the different molecular subsets, we suggest that treatment using complement inhibitors could still be rational in early stages of disease in patients with lymphoid-predominant RA while these therapies are likely to be less effective in patients with established synovitis, particularly with high degrees of TNF-driven fibroblast proliferation and myeloid inflammation.

One limitation in our study is the quantification of disease in only female mice. Sex differences have been documented and acknowledged in autoimmune diseases, including RA and SSc-PAH [61–65]. The TNF-Tg mouse model has been established to have a sexual dimorphism in its arthritis and pulmonary disease [26], with females having significantly earlier onset and more severe disease. For this study, we focused on the female mice due to the earlier onset and severity of joint and lung disease. In the study of complement deficiency in CIA mice, male mice were used and found to have ameliorated disease [17]. Therefore, examination of male TNF-Tg mice may be appropriate to fully assess if there is a difference in complement effect.

In this work, we demonstrate that the protective effect seen with a loss of complement activation through C3 or fB previously described in acute models of arthritis is largely absent in the TNF-Tg model. We also show that complement does not have a significant role in the pathology of the TNF model of pulmonary hypertension. Interestingly, there may be a protective effect of complement with regard to bone erosive disease. Overall, these results indicate that while loss of function of complement is not sufficient to overcome TNF-mediated chronic inflammation driving arthritis and PH, complement signaling may mediate erosive disease.

## Supporting information

S1 Fig Complement genes are differentially expressed between TNF- and TNF+ synovium and lung cell populations. Synovium and lung tissue were harvested from TNF- and TNF+ mice and underwent single-cell RNA-sequencing. **(A and C)** Uniform manifold approximation and projection (UMAP) plots shows the major cell populations in the synovium and lungs between TNF- and TNF+ mice. **(B and D)** Bar graphs illustrate the relative contribution of cells from TNF+ mice (in blue) versus the TNF- mice (in red) within each cell population. **(E)** Synovial fibroblasts were re-clustered to show two major fibroblast populations, lining fibroblasts and sub-lining fibroblasts, between TNF- and TNF+ mice. **(F)** Synovial fibroblasts were identified by using markers *Pdpn* and *Pdgfra*. Sub-lining fibroblasts were identified by expression of the *Thy1* marker and lining fibroblasts were identified by expression of the *Prg4* marker. **(G and H)** Expression of the complement genes *C3* and *Cfb* were differentially expressed in lining and sub-lining fibroblasts between TNF- and TNF+ mice. **(I and J)** Additionally, *C3* was differentially expressed between TNF- and TNF+ myeloid, epithelial, endothelial, and lymphocyte cells of the lung, and *Cfb* was differentially expressed in the myeloid, epithelial, and lymphocyte cells.
(TIFF)

S2 Fig Synovial fibroblast markers determine lining and sub-lining fibroblasts. **(A)** A UMAP illustrates the clusters in the TNF- and TNF+ synovial fibroblast dataset. **(B-G)** Feature plots show the expression of lining fibroblast markers *Prg4, Clic5*, *Itga6,* and *Tspan15* and expression of the sub-lining fibroblast markers, *Cd34* and *Thy1* in the TNF- and TNF+ datasets. **(H)** Feature plots also show the expression of *Rspo2* and *Col22a1*, markers associated with Prg4 + synovial lining fibroblasts. **(I-L)** Raw counts from the dataset and the initial synovium dataset were analyzed for *Cdh5*, *Clic5*, *Rspo2*, and *Col22a1* expression, illustrating an overlap of *Cdh5* and *Clic5*.
(TIFF)

S3 Fig Expression of synovial cell markers. **(A)** A UMAP illustrates the different cell clusters in the synovium. **(B)** Feature plots show the expression of marker genes that were used to determine the cell types in each cluster. *Cdh5* is a marker for endothelial cells, *Cdh1* is an epithelial cell marker, *Myf5* is a muscle cell marker, *Cd3d* and *Gata3* are T cell markers, *Cd79a* is a B cell marker, *Fcer1g* is a myeloid cell marker, and *Pdgfra* is a synovial fibroblast cell marker.
(TIFF)

S4 Fig Expression of lung cell markers. **(A)** A UMAP illustrates the cell clusters in the lung. **(B)** Feature plots show the expression of marker genes for each cluster. *Cdh1* is an epithelial cell marker, *Cd3e* is a T cell marker, *Cd79a* is a B cell marker, *Gzma* is a lymphocyte marker, *Fcer1g* is a myeloid cell marker, *Col1a2* is a mesenchyme marker, and *Cdh5* is an endothelial cell marker.
(TIFF)
