# Supplementary figures and images for: Mixed effects of complement in a chronic murine model of inflammatory erosive arthritis and pulmonary vascular disease
Source: PLoS One. 2026 Feb 5;21(2):e0340677. doi: 10.1371/journal.pone.0340677 (PMC12875489; doi:10.1371/journal.pone.0340677)

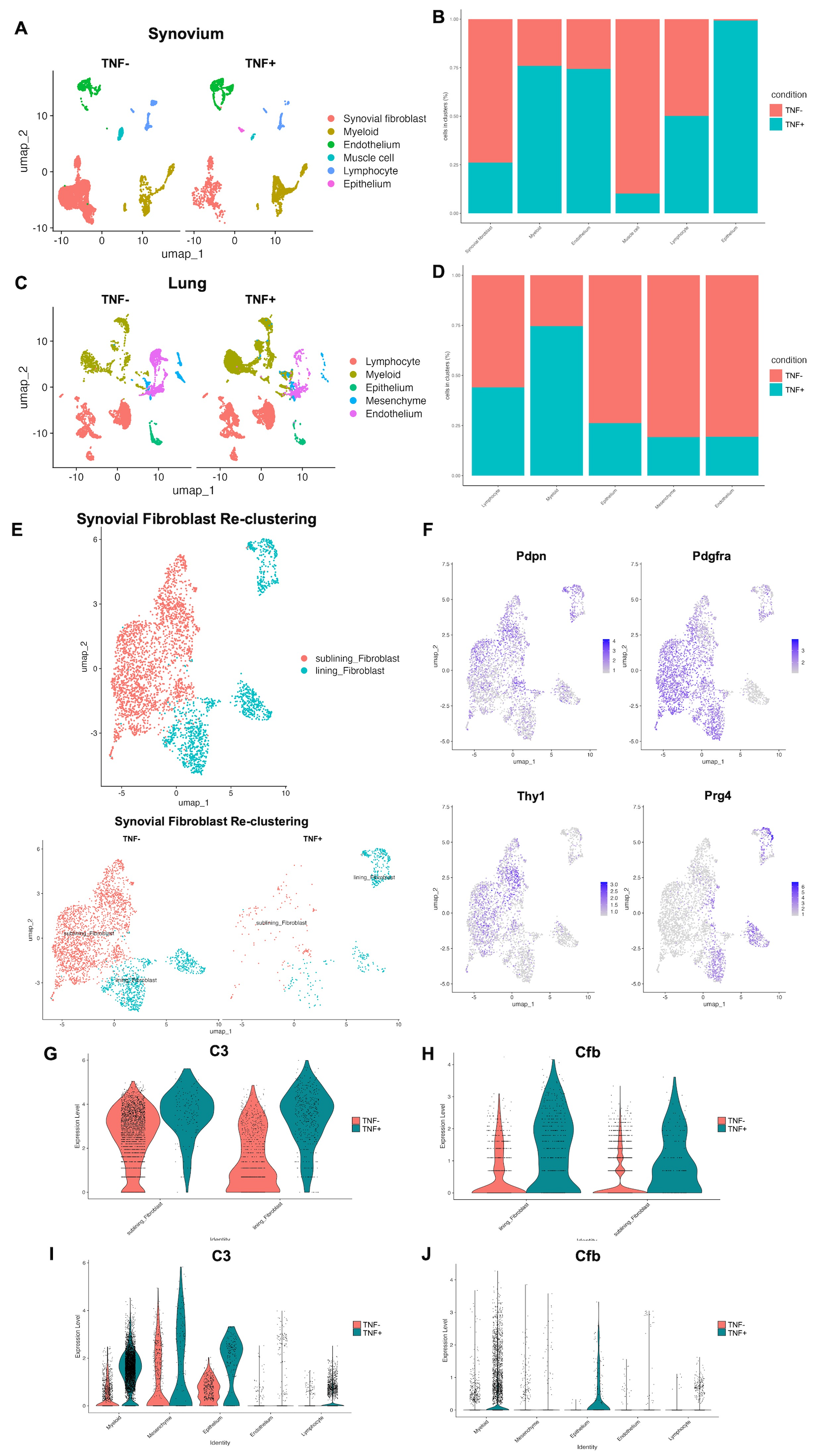

Supplement: S1 Fig — Synovium and lung tissue were harvested from TNF- and TNF+ mice and underwent single-cell RNA-sequencing. (A and C) Uniform manifold approximation and projection (UMAP) plots shows the major cell populations in the synovium and lungs between TNF- and TNF+ mice. (B and D) Bar graphs illustrate the relative contribution of cells from TNF+ mice (in blue) versus the TNF- mice (in red) within each cell population. (E) Synovial fibroblasts were re-clustered to show two major fibroblast populations, lining fibroblasts and sub-lining fibroblasts, between TNF- and TNF+ mice. (F) Synovial fibroblasts were identified by using markers Pdpn and Pdgfra. Sub-lining fibroblasts were identified by expression of the Thy1 marker and lining fibroblasts were identified by expression of the Prg4 marker. (G and H) Expression of the complement genes C3 and Cfb were differentially expressed in lining and sub-lining fibroblasts between TNF- and TNF+ mice. (I and J) Additionally, C3 was differentially expressed between TNF- and TNF+ myeloid, epithelial, endothelial, and lymphocyte cells of the lung, and Cfb was differentially expressed in the myeloid, epithelial, and lymphocyte cells. (TIFF) [file pone.0340677.s001.tiff]

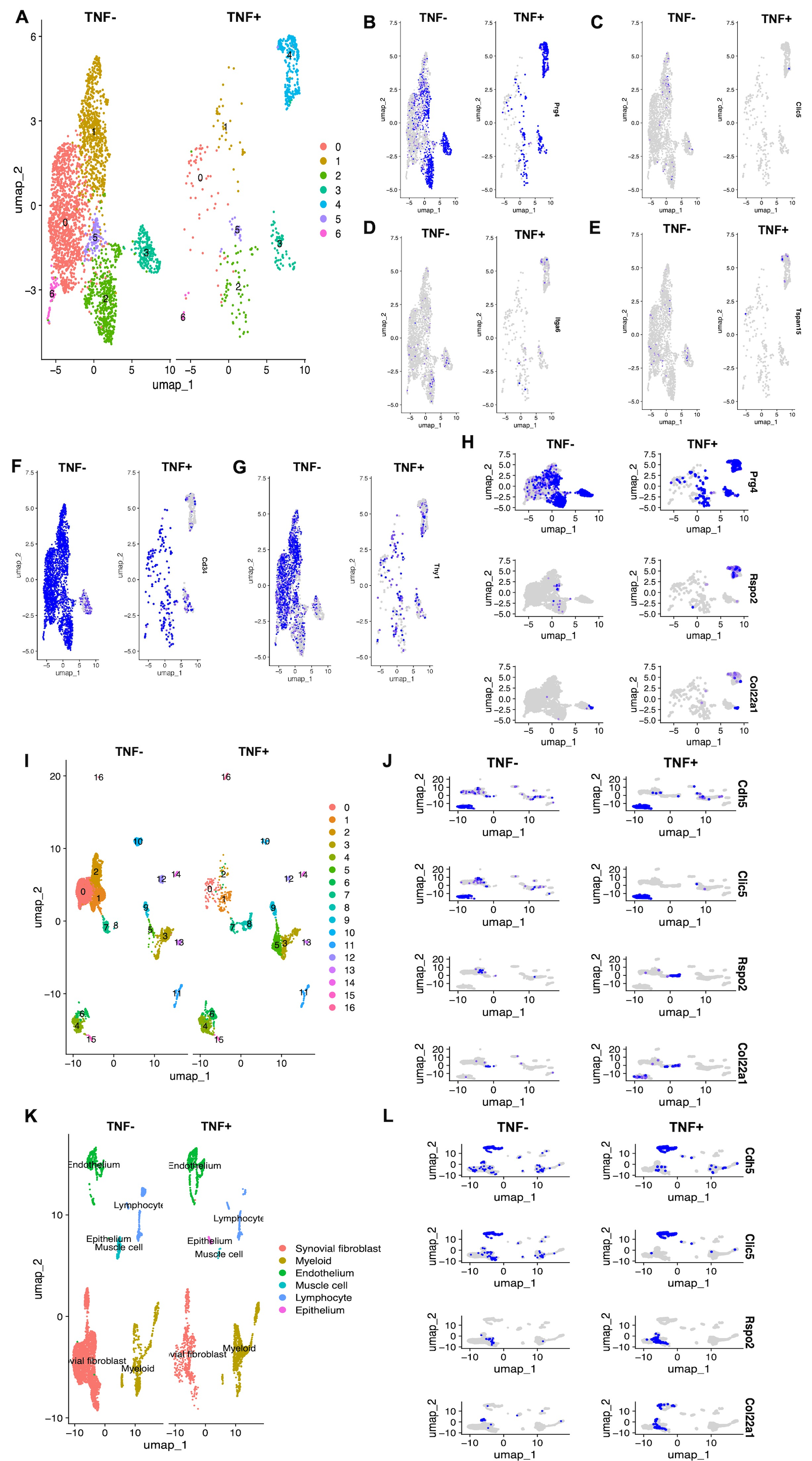

Supplement: S2 Fig — (A) A UMAP illustrates the clusters in the TNF- and TNF+ synovial fibroblast dataset. (B-G) Feature plots show the expression of lining fibroblast markers Prg4, Clic5, Itga6, and Tspan15 and expression of the sub-lining fibroblast markers, Cd34 and Thy1 in the TNF- and TNF+ datasets. (H) Feature plots also show the expression of Rspo2 and Col22a1, markers associated with Prg4 + synovial lining fibroblasts. (I-L) Raw counts from the dataset and the initial synovium dataset were analyzed for Cdh5, Clic5, Rspo2, and Col22a1 expression, illustrating an overlap of Cdh5 and Clic5. (TIFF) [file pone.0340677.s002.tiff]

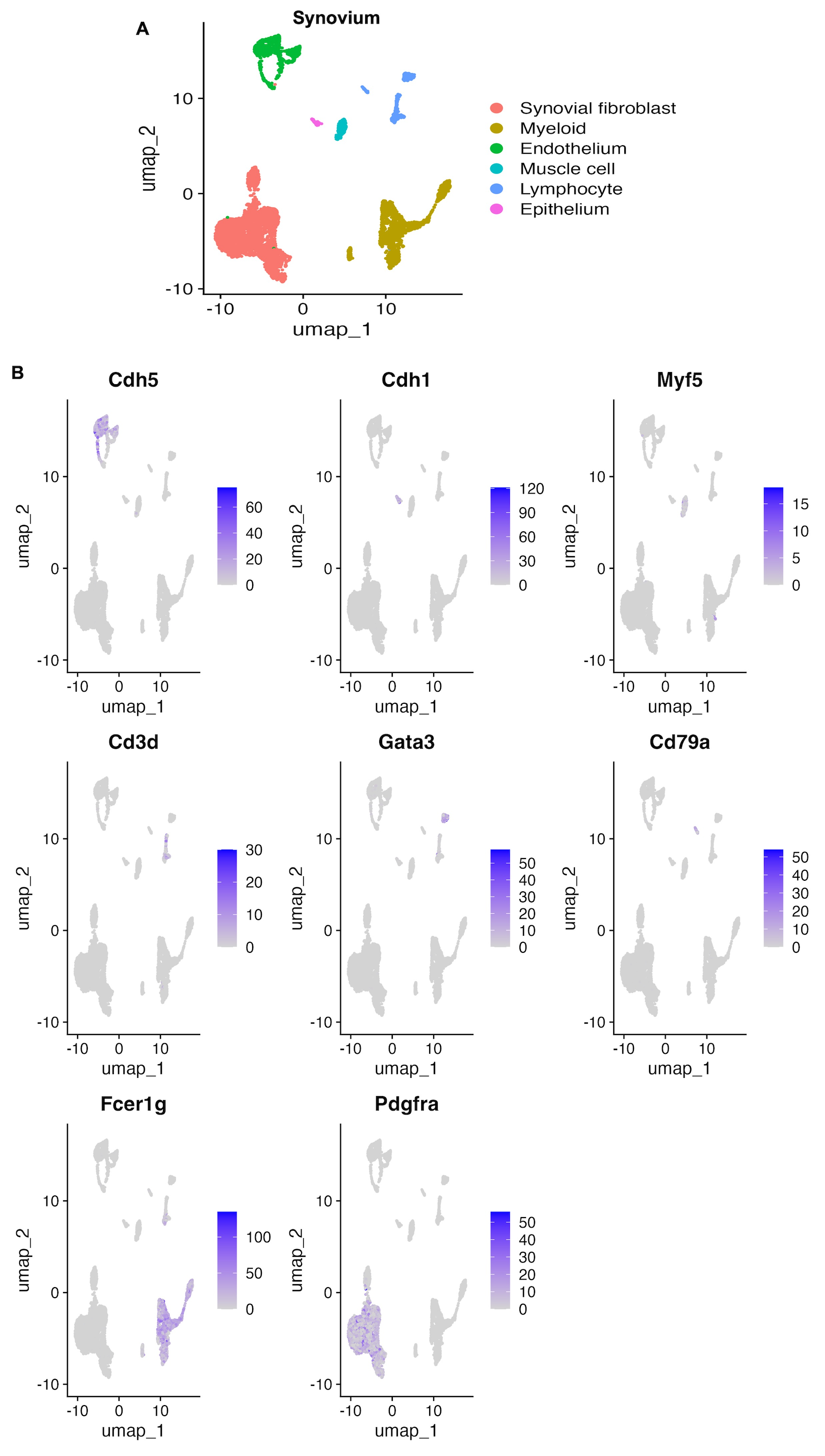

Supplement: S3 Fig — (A) A UMAP illustrates the different cell clusters in the synovium. (B) Feature plots show the expression of marker genes that were used to determine the cell types in each cluster. Cdh5 is a marker for endothelial cells, Cdh1 is an epithelial cell marker, Myf5 is a muscle cell marker, Cd3d and Gata3 are T cell markers, Cd79a is a B cell marker, Fcer1g is a myeloid cell marker, and Pdgfra is a synovial fibroblast cell marker. (TIFF) [file pone.0340677.s003.tiff]

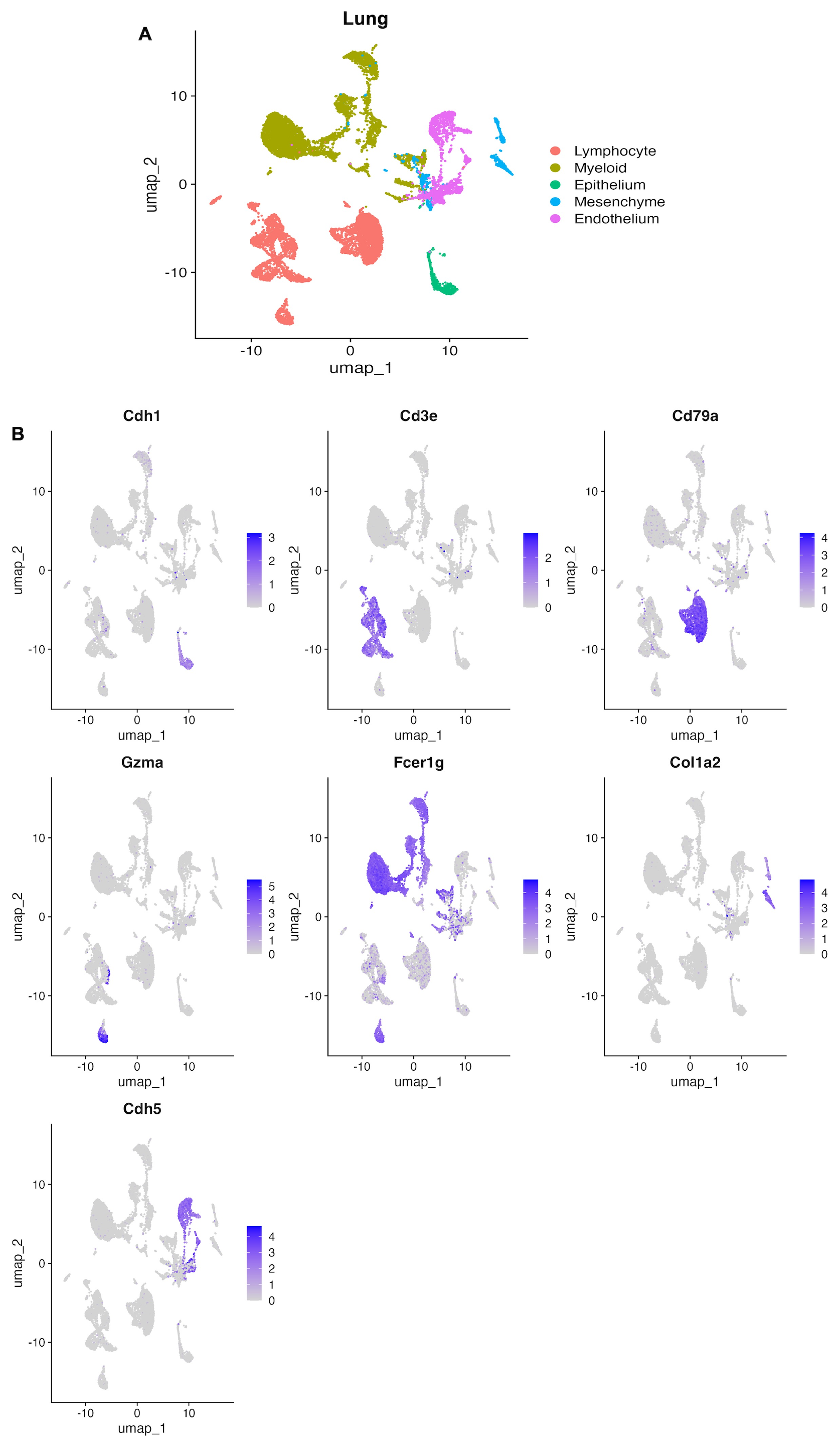

Supplement: S4 Fig — (A) A UMAP illustrates the cell clusters in the lung. (B) Feature plots show the expression of marker genes for each cluster. Cdh1 is an epithelial cell marker, Cd3e is a T cell marker, Cd79a is a B cell marker, Gzma is a lymphocyte marker, Fcer1g is a myeloid cell marker, Col1a2 is a mesenchyme marker, and Cdh5 is an endothelial cell marker. (TIFF) [file pone.0340677.s004.tiff]
